# Chronic Myeloid Leukemia: Part I—Real-World Treatment Patterns, Healthcare Resource Utilization, and Associated Costs in Later Lines of Therapy in the United States

**DOI:** 10.36469/001c.36975

**Published:** 2022-08-04

**Authors:** Ehab L. Atallah, Rodrigo Maegawa, Dominick Latremouille-Viau, Carmine Rossi, Annie Guérin, Eric Q. Wu, Pallavi Patwardhan

**Affiliations:** 1 Medical College of Wisconsin, Milwaukee; 2 Novartis Pharmaceuticals Corporation, East Hanover, New Jersey; 3 Analysis Group, Inc, Montréal, Québec, Canada; 4 Analysis Group, Inc, Boston, Massachusetts

**Keywords:** chronic myeloid leukemia, costs, economic burden, healthcare resource utilization, Medicare, treatment patterns, tyrosine kinase inhibitors

## Abstract

**Background:** Despite advances in tyrosine kinase inhibitor (TKI) therapy for chronic myeloid leukemia in chronic phase (CML-CP), a sizeable proportion of patients with CML-CP remains refractory or intolerant to these agents.

**Objectives:** Treatment patterns, healthcare resource utilization (HRU), and costs were evaluated among patients with CML who received third or later lines of therapy (3L+), a clinical population that has not been previously well-studied, with unmet treatment needs as TKI therapy has repeatedly failed.

**Methods:** Adult patients with CML who received 3L+ were identified in the IBM® MarketScan® Databases (January 1, 2001–June 30, 2019) and the SEER-Medicare–linked database (January 1, 2006–December 31, 2016). Treatment patterns were observed from CML diagnosis. HRU and direct healthcare costs (payer’s perspective, 2019 USD) were measured in a 3L+ setting.

**Results:** Among 296 commercially insured patients with 3L+ (median age, 58.5 years; female, 49.7%), the median duration of first-line (1L), second-line (2L), and 3L therapy was 8.5, 4.2, and 8.3 months, respectively. The annual incidence rate during 3L+ was 3.4 for inpatient days, 30.8 for days with outpatient services, and 1.2 for emergency department visits. Mean per-patient-per-month (PPPM) total healthcare costs (pharmacy + medical costs) were \$18 784 in 3L+, \$15 206 in 3L, and \$19 546 in 4L, with inpatient costs driving most of the difference between 3L and 4L (mean [3L] = \$2528 PPPM, mean [4L] = \$6847 PPPM). Among 53 Medicare-insured patients with 3L+ (median age, 72.0 years; female, 39.6%), the median duration of 1L, 2L, and 3L therapy was 9.7, 5.0, and 7.0 months, respectively. During 3L+, the annual incidence rate was 10.3 for inpatient days, 61.9 for days with outpatient services, and 1.5 for emergency department visits. Mean PPPM total healthcare costs were \$14 311 in 3L+, \$15 100 in 3L, and \$16 062 in 4L.

**Discussion:** Patients with CML receiving 3L+ rapidly cycled through multiple lines. Costs increased from 3L to 4L; in commercially insured patients, inpatient costs were responsible for most of the cost increase between 3L and 4L, underlying these patients’ continued need for care.

**Conclusions:** These findings support the need for better treatment options in patients with CML undergoing later lines of therapy.

## BACKGROUND

Chronic myeloid leukemia (CML) is a myeloproliferative disorder of stem cell origin that accounted for 14% of all incident leukemia cases in the United States in 2020.^1,2^ More than 95% of CML cases are characterized by a reciprocal translocation involving chromosomes 9 and 22.^2,3^ The resulting abnormally short chromosome 22, known as the Philadelphia chromosome (Ph), carries the *BCR-ABL1* gene fusion which drives the aberrant proliferation of leukemic cells.^2^

The *BCR-ABL1* gene fusion is susceptible to drug targeting by tyrosine kinase inhibitors (TKIs), which are the standard of care for patients with CML in all phases, although patients are typically diagnosed in the chronic phase (ie, CML-CP).^2^ The introduction of the first-generation (1G) TKI imatinib 2 decades ago provided a paradigm shift that markedly improved patients’ prognosis.^4–6^ However, 25% to 30% of patients in chronic phase treated in a clinical trial setting developed resistance to imatinib after nearly 5 years of treatment.^7,8^ Resistance occurs through the emergence of mutations that prevent the binding of TKI to the *ABL1* kinase domain.^9^ More efficacious second-generation (2G) TKIs, including dasatinib, nilotinib, and bosutinib, were subsequently developed and initially approved for patients with resistance or intolerance to prior therapy, including imatinib^9–12^; their approval has since been expanded to patients with newly diagnosed Ph CML^13,14^ More recently, the third-generation (3G) TKI ponatinib was approved in patients intolerant or resistant to at least 2 TKIs and in patients whose leukemias express the T315I mutation of BCR-ABL1.^15^ For patients who present or progress to accelerated phase or blast crisis and/or are resistant or intolerant to multiple TKIs, allogeneic hematopoietic stem cell transplantation (HSCT), which was previously used for CML-CP treatment, remains a viable option.^2,16^

Despite this remarkable progress, a number of patients with CML-CP cycle through multiple therapies and undergo a third or later lines of therapy (3L+).^17–19^ However, scant data are available on the treatment patterns of patients initiating a 3L+ in the real-world clinical practice setting. Similarly, the economic outcomes of these patients are not well documented.

A better understanding of these outcomes may provide important insights on how new treatments may address the unmet needs and financial burden of patients for whom currently available TKI therapies repeatedly fails. Therefore, this study was conducted to evaluate treatment patterns, healthcare resource utilization (HRU), and healthcare costs in patients with CML who received a 3L+.

## METHODS

### Data Sources

Two health insurance claims databases were used to conduct this study: the IBM® MarketScan® Commercial Claims and Encounters and Medicare Supplemental Databases (commercially insured population; January 1, 2001–June 30, 2019), and the Surveillance Epidemiology, and End Results (SEER)-Medicare–linked database (Medicare-insured population; January 1, 2006–December 31, 2015, for the SEER cancer registry component and January 1, 2007–December 31, 2016, for the linked Medicare claims component).

MarketScan® is a large commercial claims database that covers all census regions in the United States.^20^ This database contains detailed claims information from approximately 350 payers, representing approximately 51 million covered lives in the most recent full data year. Information on history of health plan enrollment, demographics, diagnoses, claims for medical care received across all settings, and claims for pharmacy services are available for covered employees and their dependents.

The SEER-Medicare database is provided by the US National Institutes of Health–National Cancer Institute and is composed of 2 large databases linked at the patient level: (1) the SEER database of the National Cancer Institute, which contains data on cancer cases diagnosed from 1973 through 2015, and (2) the Medicare claims database (Parts A, B, and D; available from 2006 through 2016; **Supplementary Figure S1**).^21^ The SEER database includes demographic data and detailed clinical information on cancer site, stage, and histology. The Medicare database includes claims related to hospital care (ie, hospital, skilled nursing facility, hospice, and some home health care; Part A), outpatient and physician medical services, durable medical equipment use, clinical research, ambulance services, and rehabilitation services (Part B), and outpatient drug prescriptions (Part D).

All analyses were conducted separately in these 2 distinct databases to provide both payer perspectives, without any comparison between the commercial and Medicare populations due to inherent database and population differences. The SEER-Medicare analysis received an institutional review board (IRB) exemption from the Western Copernicus Group Institutional Review Board. Data from both databases are de-identified and comply with the confidentiality requirements of the Health Insurance Portability and Accountability Act (HIPAA). Per data user agreement, due to the cell suppression policy of the Centers for Medicare & Medicaid Services, cell values of less than 11 cannot be displayed for results from SEER-Medicare data.

### Study Design and Setting

A retrospective cohort study was used. The observation period spanned from the first observed CML diagnosis to the end of continuous plan enrollment or end of data availability, whichever came first (**Supplementary Figure S2**). The index date was defined as the date of initiation of the third line (3L) of therapy. The baseline period was defined as the 6-month period preceding the index date, with patient characteristics measured during this period. To accurately capture all lines of CML therapy, the study design included a washout period of at least 6 months before the first observed line to ensure that patients had no prior CML therapy, which was determined based on clinical input and was previously used in retrospective, claims-based analyses for CML to identify first-line (1L) therapy.^22–25^

Pharmacy and medical claims for the following treatments were considered to identify lines of therapy for CML-CP during the observation period, conditional on Food and Drug Administration approval dates: bosutinib (September 4, 2012, for CML-CP with resistance/intolerance to prior therapy; December 19, 2017, for newly diagnosed CML-CP,^11,26^ dasatinib (June 28, 2006, for CML-CP with resistance/intolerance to prior therapy; October 28, 2010, for newly diagnosed CML-CP^10,27^), imatinib (May 10, 2001, for CML-CP after interferon alfa failure^5^), nilotinib (October 29, 2007, for CML-CP with resistance/intolerance to prior therapy; June 17, 2010, for newly diagnosed CML-CP,^12,28^ ponatinib (December 14, 2012, for CML-CP with resistance/intolerance to prior TKIs^29^), and omacetaxine mepesuccinate (October 26, 2012, for CML-CP with resistance and/or intolerance to ≥2 TKIs^30^). Each line started at the first claim for a given treatment and ended upon one of the following events: switch to another CML-CP treatment, initiation of a chemotherapy not listed above for CML-CP, HSCT procedure, treatment discontinuation (gap of ≥90 consecutive days; if the same treatment was resumed after the treatment gap, this was considered as noncompliance/treatment interruption rather than a separate line), and end of observation period. Hydroxyurea was only considered as a pretreatment for CML-CP.

The follow-up period was defined from 3L initiation to the earliest of end of data availability, end of continuous health plan enrollment, HSCT, or chemotherapy not listed in CML-CP treatment.

### Patient Selection Criteria

**Figure 1.**
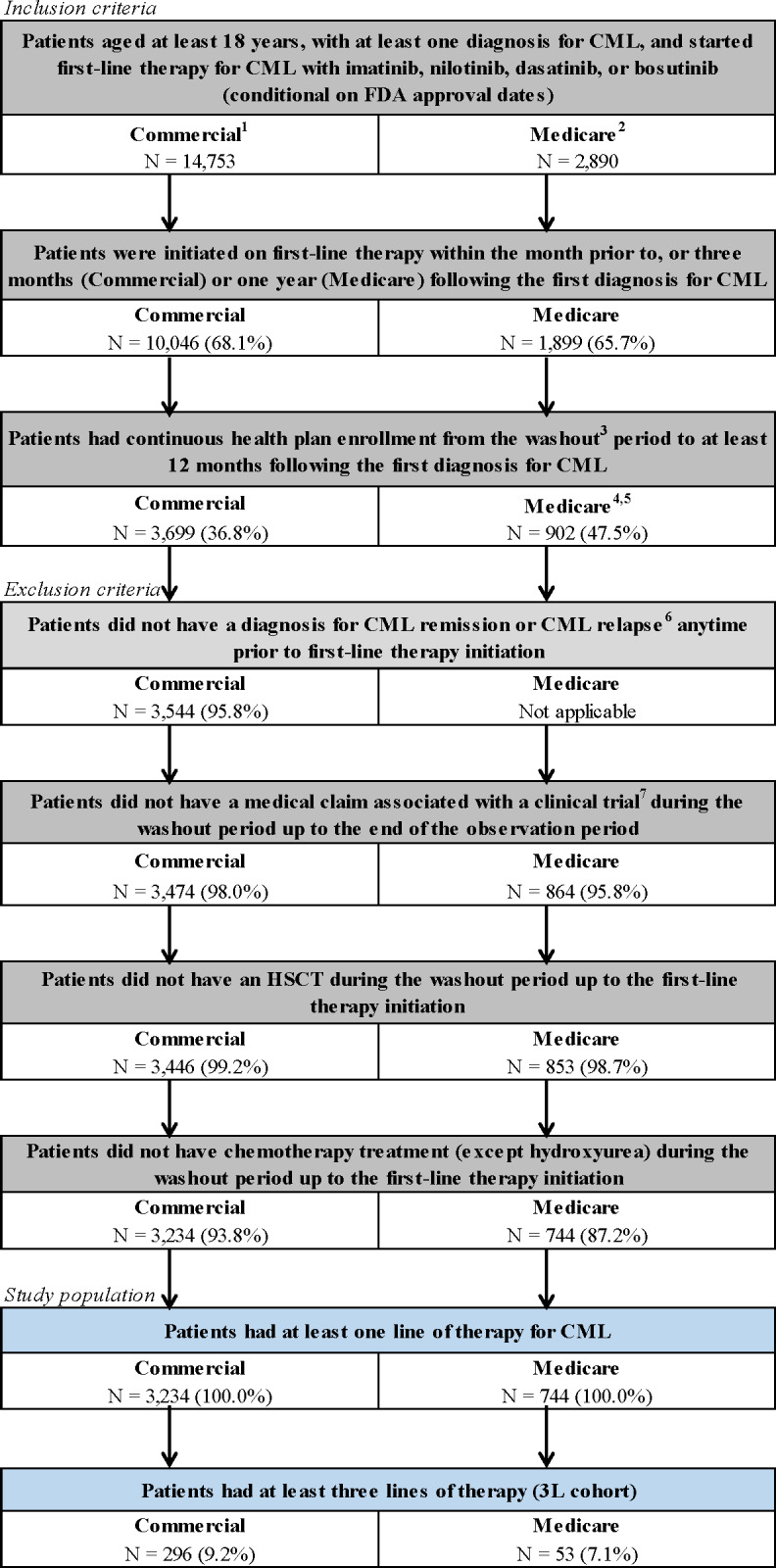
Patient Selection Flowchart Abbreviations: 3L, third line; CML, chronic myeloid leukemia; FDA, US Food and Drug Administration; HSCT, hematopoietic stem cell transplant; ICD-9-CM, *International Classification of Diseases, Ninth Revision Clinical Modification*; ICD-10-CM, *International Classification of Diseases, Tenth Revision Clinical Modification*; ICD-O-3; *International Classification of Diseases for Oncology, Third Edition*; Ph, Philadelphia chromosome–positive. ^1^Patients had at least 1 diagnosis for CML (ICD-9-CM: 205.1x, ICD-10-CM: C92.1x), with first CML diagnosis observed in claims on or after May 10, 2001, the date of FDA approval for imatinib. ^2^Patients had at least 1 diagnosis for Ph+ CML (ICD-O-3: 9863 or 9875 or site recode 35022). ^3^The washout period was defined as a period of at least 6 months prior to first-line therapy initiation for CML. ^4^For Medicare, patients were excluded if they had HMO coverage (ie, Medicare Part C) during their continuous health plan enrollment. ^5^For Medicare, the 12-month requirement following CML diagnosis did not apply to those patients who died. ^6^CML remission was defined using ICD-9-CM code 205.11 or ICD-10-CM code C92.11; CML relapse was defined using ICD-9-CM code 205.12 or ICD-10-CM code C92.12. ^7^Medical claim associated with a clinical trial was defined using ICD-9-CM code V70.7 or ICD-10-CM code Z00.6. For Medicare, clinical trial was also defined using services provided during a clinical trial in the NCH file under the CLN_TRIL variable.

For the analysis of the commercially insured population, adult patients with at least 1 CML diagnosis (*International Classification of Diseases, Ninth Revision, Clinical Modification* code: 205.1x; *International Classification of Diseases, Tenth Revision, Clinical Modification* code: C92.1x) who initiated 1L CML-CP therapy with bosutinib, dasatinib, imatinib, or nilotinib, were identified (Figure 1; conditional on Food and Drug Administration approval date). For the Medicare-insured population, adult patients with a confirmed Ph CML diagnosis (*International Classification of Diseases for Oncology, Third Edition* codes: 9863, 9875, or site recode 35022) who initiated 1L CML-CP therapy listed above were identified (Figure 1). For both populations, patients were required to initiate 1L therapy within the month prior to, or 3 months (commercial) or 1 year (Medicare) following the first CML diagnosis and had continuous health plan enrollment from the washout period to at least 12 months following the first diagnosis for CML. Detailed inclusion and exclusion criteria are provided in Figure 1. The analytical sample included all patients who initiated at least 3 lines of therapy (ie, 3L+) and treatment sequences to reach 3L+ were summarized.

### Outcomes and Statistical Analyses

Among patients who reached later lines of therapy (ie, 3L+), treatment patterns, including the CML-CP treatment used, duration of the line of therapy, and description of the event that defined the end of the line, were described from 1L to the fourth line of therapy (4L).

In addition, all-cause and CML-related HRU were assessed for inpatient (IP) admissions and days, days with outpatient (OP) services, and emergency department (ED) visits. HRU outcomes were reported using annual incidence rates (IR). Total all-cause and CML-related costs were broken down into pharmacy and medical costs, with medical costs further stratified into IP, OP, and ER costs. All costs were reported per patient per month (PPPM) from the payers’ perspectives (ie, paid by the health plan and coordination of benefits for commercially insured population; paid by Medicare and coinsurers [Medicaid] for the Medicare-insured population). Costs were inflated to 2019 US dollars based on the medical care component of the consumer price index.^31^ Costs of HSCT were included in the main analysis, and event-level costs of HSCT in the IP setting was reported separately. A sensitivity analysis in which HSCT costs were excluded was also conducted.

Healthcare resource utilization and cost outcomes were evaluated during 3 periods: (1) 3L+ (from 3L initiation to the end of the follow-up period including later lines of therapy), (2) during 3L (from 3L initiation to end of 3L therapy), and (3) during 4L (from 4L initiation to end of 4L therapy). CML-related HRU and costs were identified using medical service claims with a diagnosis for CML or medical service claims with a procedure code for omacetaxine. CML-related pharmacy costs were identified using prescriptions filled for a TKI.

All analyses were descriptive. Continuous variables, including healthcare costs, were summarized using means, medians, and SD; categorical variables were summarized using frequencies and counts. Analyses were conducted using SAS Enterprise Guide software version 7.1 (SAS Institute, Cary, North Carolina).

## RESULTS

### Sample Selection

**Figure 2.**
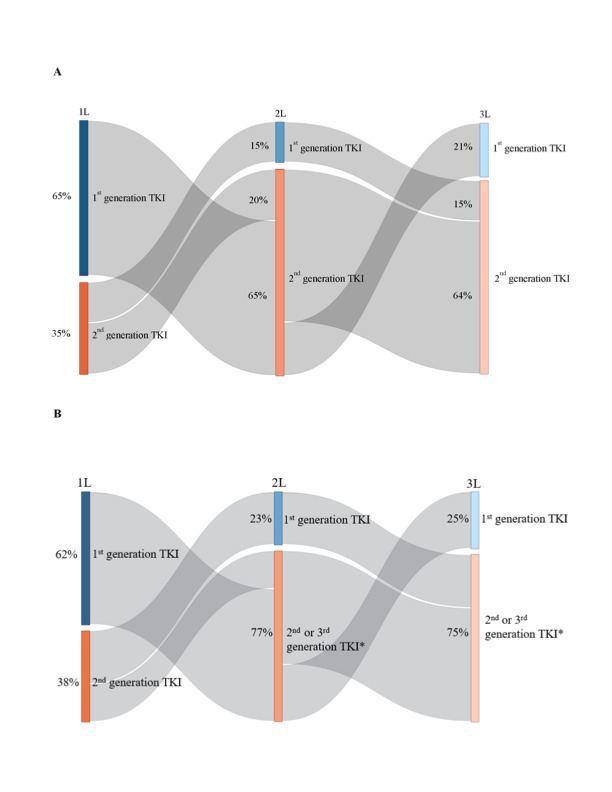
Treatment Sequences Observed Between the 1L and 3L Therapy **(A)** Among Commercially Insured Patients and **(B)** Among Medicare Beneficiaries Abbreviations: 1L, first line; 2L, second line; 3L, third line; TKI, tyrosine kinase inhibitor. *Ponatinib was rare; it was not used in 1L and only a few patients received ponatinib in 2L or 3L.

**Commercially insured population:** In the commercially insured population, from January 1, 2001, to June 30, 2019, 3234 patients were observed to receive a 1L for CML (Figure 1). Of these, 954 (29.5%) were observed to receive a 2L, 296 (9.2%) were observed to receive a 3L, 83 (2.6%) were observed to receive a 4L, 24 (0.7%) were observed to receive a fifth line of therapy (5L), and 10 (0.3%) were observed to receive a sixth line (6L). Among those observed with at least a 2L (n=954), most of the patients cycled through imatinib and dasatinib in 1L and 2L (54.1%); this was consistent among a more contemporary subgroup of patients with CML treatments after the introduction of the generic version of imatinib in 2016 (n=208; 48.6%). Based on TKI grouping, 65.7% received a 1G TKI and 34.3% a 2G TKI in 1L, and 15.0% received a 1G TKI and 83.9% a 2G TKI in 2L (with the remainder using a 3G TKI). These patterns of treatment sequences were also consistent among those with at least a 3L (n=296), with most of the patients cycling through imatinib and dasatinib in 1L and 2L before reaching the 3L (51.4%; 43.9% among those with CML treatment after introduction of imatinib generic version [n=57]), where 64.9% received a 1G TKI and 35.1% a 2G TKI in 1L, and 15.2% received a 1G TKI and 84.5% a 2G TKI in 2L (with the remainder using a 3G TKI; Figure 2A).

**Medicare-insured population:** In the Medicare-insured population, from January 1, 2007, to December 31, 2016, 744 patients were observed to receive a 1L for CML (Figure 1). Of these, 213 (28.6%) were observed to receive a 2L, 53 (7.1%) were observed to receive a 3L, and 12 (1.6%) were observed to receive a 4L. Few patients were observed to receive a 5L. Among those observed with at least a 2L (n=213), most of the patients cycled through imatinib and dasatinib in 1L and 2L (55.4%). Based on TKI grouping, 59.2% received a 1G TKI and 40.8% a 2G TKI in 1L, and 24.9% received a 1G TKI and 75.1% a 2G/3G TKI in 2L (ponatinib was rare in the 2L). These patterns of CML treatment sequences were also consistent among those with at least a 3L (n=53), with most of the patients cycling through imatinib and dasatinib in 1L and 2L before reaching the 3L (54.7%), where 62.3% received a 1G TKI and 37.7% a 2G TKI in 1L, and 22.6% received a 1G TKI and 77.4% a 2G/3G TKI in 2L (ponatinib was rare in the 2L; Figure 2B).

### Baseline Characteristics of Patients Receiving 3L+

**Commercially insured population**: Among commercially insured patients observed with at least a 3L (n = 296), 49.7% of patients were female. At the time of 3L initiation, median age was 58.0 years, with 29.7% who were aged at least 65 years (Table 1). At baseline, the mean modified Charlson Comorbidity Index score (CCI) (excluding CML) was 1.6, including 24.0% of patients who had a score of at least 3. The most prevalent comorbidities were hypertension (45.3%) and diabetes (25.0%). In total, 64.2% of patients had moderate or severe disease based on the Darkow disease complexity index. Overall, 20.6% of patients were likely unfit for HSCT due to age at least 75 years or having congestive heart failure, cirrhosis, or end-stage renal disease.

**Table 1.** Patient Baseline Characteristics

**Patient Characteristics**	**Commercial (n=296)**	**Medicare (n=53)^a^**
At 3L initiation		
Demographic characteristics		
Age, years; mean ± SD [median]	58.52±13.88 [58.00]	70.34±12.12 [72.00]
<65 years, n (%)	208 (70.3)	NA
65+ years, n (%)	88 (29.7)	NA
Female, n (%)	147 (49.7)	21 (39.6)
White (race/ethnicity),^b^ n (%)	—	44 (83.0)
Census region of residence,^c^ n (%)		
South	120 (40.5)	16 (30.2)
Midwest/North Central	81 (27.4)	NA
Northeast	44 (14.9)	NA
West	51 (17.2)	19 (35.8)
Commercial health plan type^d^, n (%)		
Preferred provider organization	148 (50.0)	—
Medicare supplemental coverage	89 (30.1)	—
Comprehensive	56 (18.9)	—
Home maintenance organization	43 (14.5)	—
Other	44 (14.9)	—
Unknown	5 (1.7)	—
Calendar year of first CML diagnosis,^e^ n (%)		
2001-2004	9 (3.0)	NA
2005-2008	55 (18.6)	NA
2009-2012	120 (40.5)	NA
2013-2018	112 (37.8)	20 (37.7)
During the 6-month period prior to specific therapy initiation		
Modified CCI, excluding CML,^f^ mean ± SD [median]	1.64±1.69 [2.00]	3.60±2.31 [3.00]
Darkow Disease Complexity Index,^g^ n (%)		
Mild	106 (35.8)	NA
Moderate	104 (35.1)	NA
Severe^h^	86 (29.1)	24 (45.3)
Use of hydroxyurea (pre-treatment), n (%)	21 (7.1)	NA
Patients likely unfit for HSCT,^i^ n (%)	61 (20.6)	34 (64.2)
Other mental and physical comorbidities^j^ (top 6 most prevalent), n (%)		
Cardiac arrhythmias	55 (18.6)	20 (37.7)
Chronic pulmonary disease	45 (15.2)	22 (41.5)
Congestive heart failure	32 (10.8)	22 (41.5)
Diabetes	74 (25.0)	25 (47.2)
Hypertension	134 (45.3)	39 (73.6)
Valvular disease	35 (11.8)	17 (32.1)
During the observation period		
Observation period duration,^k^ months; mean ± SD [median]	57.79 ± 36.31 [47.99]	58.51 ± 24.07 [56.12]

Abbreviations: 3L, third line; CCI, Charlson Comorbidity Index; CML, chronic myeloid leukemia; ESRD, end-stage renal disease; HSCT, hematopoietic stem cell transplant; NA, not available (due to CMS cell suppression policy). ^a^Few patients were eligible for Medicare via disability status in SEER-Medicare. ^b^Race/ethnicity data were only available in SEER-Medicare. ^c^For SEER-Medicare, only the 2 regions with the greatest proportions are reported. ^d^Commercial health plan types were not mutually exclusive. ^e^Most other patients received their first CML diagnosis between 2009 and 2012 in SEER-Medicare. ^f^From Quan H, Sundararajan V, Halfon P, et al. Coding algorithms for defining comorbidities in ICD-9-CM and ICD-10 administrative data. *Med Care.* 2005;43:1130-1139, and Quan H, Li B, Couris CM, et al. Updating and validating the Charlson comorbidity index and score for risk adjustment in hospital discharge abstracts using data from 6 countries. *Am J Epidemiol.* 2011;173(6):676-682. ^g^From Darkow T, Henk HJ, Thomas SK, et al. Treatment interruptions and non-adherence with imatinib and associated healthcare costs: a retrospective analysis among managed care patients with chronic myelogenous leukemia. *Pharmacoeconomics*. 2007;25(6):481-496. ^h^Most other patients had moderate disease in SEER-Medicare. ^i^Patients were considered unfit for HSCT if they met any of the following conditions: (1) were at least 75 years of age, (2) had congestive heart failure, (3) had cirrhosis, or (4) had end-stage renal disease. ^j^From Elixhauser A, Steiner C, Harris DR, Coffey RM. Comorbidity measures for use with administrative data. *Med Care*. 1998;36(1):8-27. ^k^The observation period was defined as a period of at least 12 months from the first CML diagnosis to (1) end of data availability or (2) end of health plan coverage, whichever occurred first.

**Medicare-insured population**: Among Medicare-insured patients observed with at least 3L (n = 53), median age was 72.0 years at the time of 3L initiation, and most patients were at least 65 years old; few were enrolled via disability status (Table 1). In total, 39.6% of patients were female, and 83.0% were non-Hispanic White. The mean modified CCI score (excluding CML) was 3.6, including 64.2% who had a score of at least 3. The most prevalent comorbidities were hypertension (73.6%), diabetes (47.2%), chronic pulmonary disease (41.5%), congestive heart failure (41.5%), and cardiac arrhythmias (37.7%). Most patients (64.2%) were likely unfit to undergo HSCT.

### Treatment Patterns of Patients Receiving 3L+

**Commercially insured population**: In the commercially insured population, most patients had their first CML diagnosis on or after 2009 (78.4%) and were observed over an average of 57.8 months from this CML diagnosis. Most patients initiated 3L on or after 2013 (62.8%; Table 2). The mean (median) duration of 1L, second line of therapy (2L), and 3L were 14.9 (8.5), 10.4 (4.2), and 15.6 (8.3) months, respectively; 52.0% of patients were still receiving 3L at the end of follow-up. Between 1L and 3L, only TKIs were used for CML treatment. The most common TKIs used in each line were imatinib in 1L (64.9%), dasatinib in 2L (49.0%), and nilotinib in 3L (36.1%). During 3L, 20.9% of patients received a 1G TKI, 74.7% received a 2G TKI, and 4.4% received a 3G TKI (ie, ponatinib). Eighty-three (28.0%) patients received a 4L therapy, with dasatinib being the most common TKI used during this line. Overall, 6.8% of patients received HSCT after the 3L of therapy.

**Table 2.** Treatment Patterns in 1L-4L Among Patients Receiving 3L+

**Treatment Pattern**	**1L**	**2L**	**3L**	**4L**
Commercial	n=296	n=296	n=296	n=83
Treatment received, n (%)				
Imatinib	192 (64.9)	45 (15.2)	62 (20.9)	17 (20.5)
Dasatinib	64 (21.6)	145 (49.0)	73 (24.7)	24 (28.9)
Nilotinib	40 (13.5)	92 (31.1)	107 (36.1)	12 (14.5)
Bosutinib	0 (0.0)	13 (4.4)	41 (13.9)	22 (26.5)
Ponatinib	0 (0.0)	1 (0.3)	13 (4.4)	6 (7.2)
Omacetaxine mepesuccinate	0 (0.0)	0 (0.0)	0(0.0)	2 (2.4)
Calendar year of line of therapy initiation, n (%)				
2001-2004	10 (3.4)	0 (0.0)	0 (0.0)	0 (0.0)
2005-2008	55 (18.6)	27 (9.1)	11 (3.7)	3 (3.6)
2009-2012	118 (39.9)	121 (40.9)	99 (33.4)	19 (22.9)
2013-2019	113 (38.2)	148 (50.0)	186 (62.8)	61 (73.5)
Duration of line of therapy, mo; mean ± SD [median]	14.92±18.70 [8.47]	10.43±15.19 [4.24]	15.61±18.49 [8.32]	14.34±15.43 [8.39]
One prescription fill, n (%)	31 (10.5)	73 (24.7)	45 (15.2)	13 (15.7)
≤3 mo duration, n (%)	78 (26.4)	128 (43.2)	87 (29.4)	25 (30.1)
>3 to ≤6 mo duration, n (%)	50 (16.9)	43 (14.5)	37 (12.5)	8 (9.6)
>6 mo duration, n (%)	168 (56.8)	125 (42.2)	172 (58.1)	50 (60.2)
>12 mo duration, n (%)	116 (39.2)	83 (28.0)	118 (39.9)	33 (39.8)
Treatment-free period among those with an observed subsequent line, mo; mean ± SD [median]	1.28±4.08 [0.13]	2.56±5.45 [0.44]	1.54±3.02 [0.30]	1.67±2.69 [0.07]
Event at the end of line of therapy, n (%)				
Discontinued treatment (treatment-free ≥90 days)^a^	0 (0.0)	0 (0.0)	24 (8.1)	12 (14.5)
Switch to another TKI or omacetaxine	296 (100.0)	296 (100.0)	83 (28.0)	24 (28.9)
CML-AP/BC chemotherapy	0 (0.0)	0 (0.0)	17 (5.7)	4 (4.8)
Hematopoietic stem cell transplant	0 (0.0)	0 (0.0)	18 (6.1)	1 (1.2)
Still on the line of therapy at end of data	0 (0.0)	0 (0.0)	154	42
Medicare	n = 53	n = 53	n = 53	n = 12
Treatment received,^b^ n (%)				
1G	33 (62.3)	12 (22.6)	13	NA
2G or 3G	20 (37.7)	41 (77.4)	40	NA
Omacetaxine mepesuccinate	0 (0.0)	0 (0.0)	0	0
Calendar year of line of therapy initiation,^c^ n (%)				
2013-2016	21 (39.6)	34 (64.2)	NA	12
Duration of line of therapy,^d^ mo; mean ± SD [median]	18.19 ± 20.45 [9.74]	10.03 ± 12.21 [5.00]	13.93 ± 15.41 [7.01]	13.43 ± 8.34 [11.91]
≤6 mo duration, n (%)	19(35.8)	32(60.4)	23 (43.4)	NA
>6 mo duration, n (%)	34(64.2)	21(39.6)	30 (56.6)	NA
>12 mo duration, n (%)	25 (47.2)	17 (32.1)	20 (37.7)	NA
Treatment-free period among those with an observed subsequent line, mo; mean ± SD [median]	1.56 ± 5.55 [0.23]	3.86 ± 10.14 [0.49]	2.40 ± 4.68 [0.81]	2.57 ± 3.54 [2.57]
Event at the end of line of therapy, n (%)				
Discontinued treatment (treatment-free ≥90 days)a	0 (0.0)	0 (0.0)	NA	NA
Switch to another TKI or omacetaxine	53 (100.0)	53 (100.0)	12	NA
CML-AP/BC chemotherapy	0 (0.0)	0 (0.0)	NA	NA
Hematopoietic stem cell transplant	0 (0.0)	0 (0.0)	NA	NA
Still on the line of therapy at end of data	0 (0.0)	0 (0.0)	27	NA

Abbreviations: 1G, first generation; 2G, second generation; 3G, third generation; 1L, first line; 2L, second line; 3L, third line; 4L, fourth line; AP, accelerated phase; BC, blast crisis; CML, chronic myeloid leukemia; NA, not available (due to cell suppression policy); TKI, tyrosine kinase inhibitor. ^a^Treatment discontinuation was defined as a gap of ≥90 days between the end of the last day of supply and the end of the observation period. ^b^In the 4L, most patients received 3G (ie, ponatinib). ^c^The majority of patients initiated 3L in 2013-2016. ^d^For each line of therapy, the proportions of patients with <3 months on a given line were similar to those observed among commercially insured patients.

**Medicare-insured population**: In the Medicare-insured population, the vast majority of patients received their first CML diagnosis in 2009 or later and patients were observed over an average of 58.5 months from this CML diagnosis. Almost all patients initiated 3L on or after 2013. The mean (median) durations of 1L, 2L, and 3L were 18.2 (9.7), 10.0 (5.0), and 13.9 (7.0) months, respectively; 50.9% of patients were still in their 3L at the end of follow-up (Table 2). The most commonly used 1L, 2L, and 3L TKIs were imatinib (62.3%), dasatinib (39.6%), and nilotinib (35.8%), respectively. No bosutinib use was observed in 3L+ in the Medicare-insured population, likely due to the limited sample size. During 3L, 24.5% of patients received a 1G TKI, with almost all of the remainder using a 2G TKI; few patients received a 3G TKI (ie, ponatinib). Overall, 12 (22.6%) patients went on to receive a 4L therapy, with ponatinib being the most commonly used treatment option during this line. HSCT was rare in later lines in this population.

### Healthcare Resource Utilization and Costs of Patients Receiving 3L+

**Figure 3.**
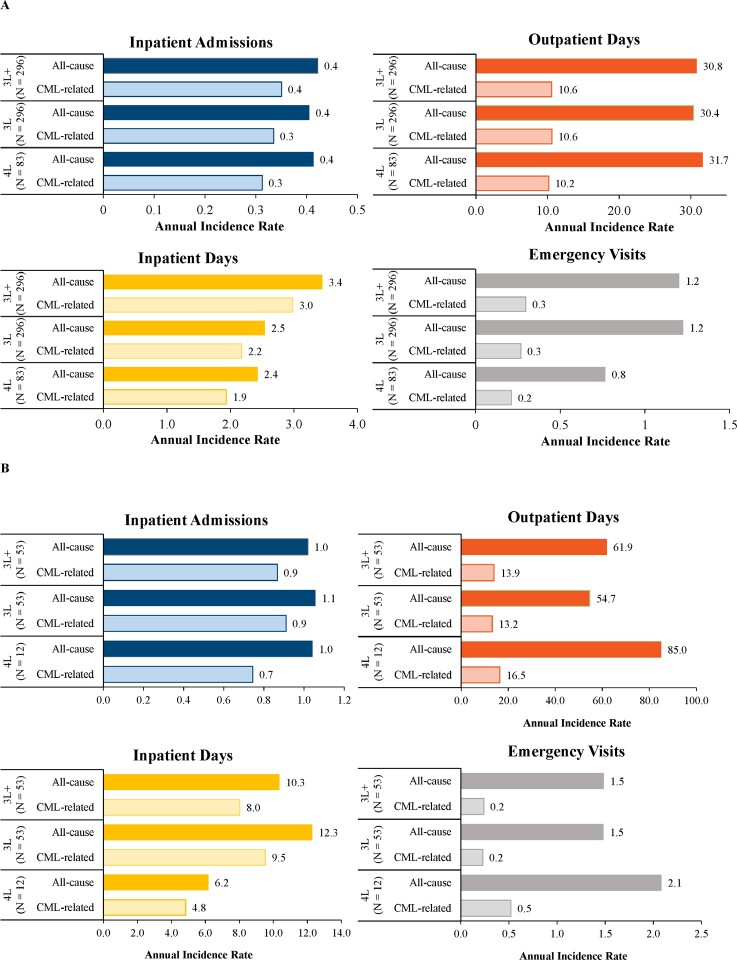
Healthcare Resource Utilization Among **(A)** Commercially Insured Patients and **(B)** Medicare-Insured Patients Abbreviations: 3L, third line; 4L, fourth line; 3L+, third line or later; CML, chronic myeloid leukemia.

**Commercially insured population:** Commercially insured patients were observed over a mean (median) follow-up of 24.5 months (17.3 months) during 3L+. Patients had 0.4 IP stays per year, of which 83.1% were CML-related (Figure 3A). During the same period, the annual IR for IP days, OP days, and ED visits were 3.4, 30.8, and 1.2, respectively. Similar results were obtained during 3L and 4L.

**Figure 4.**
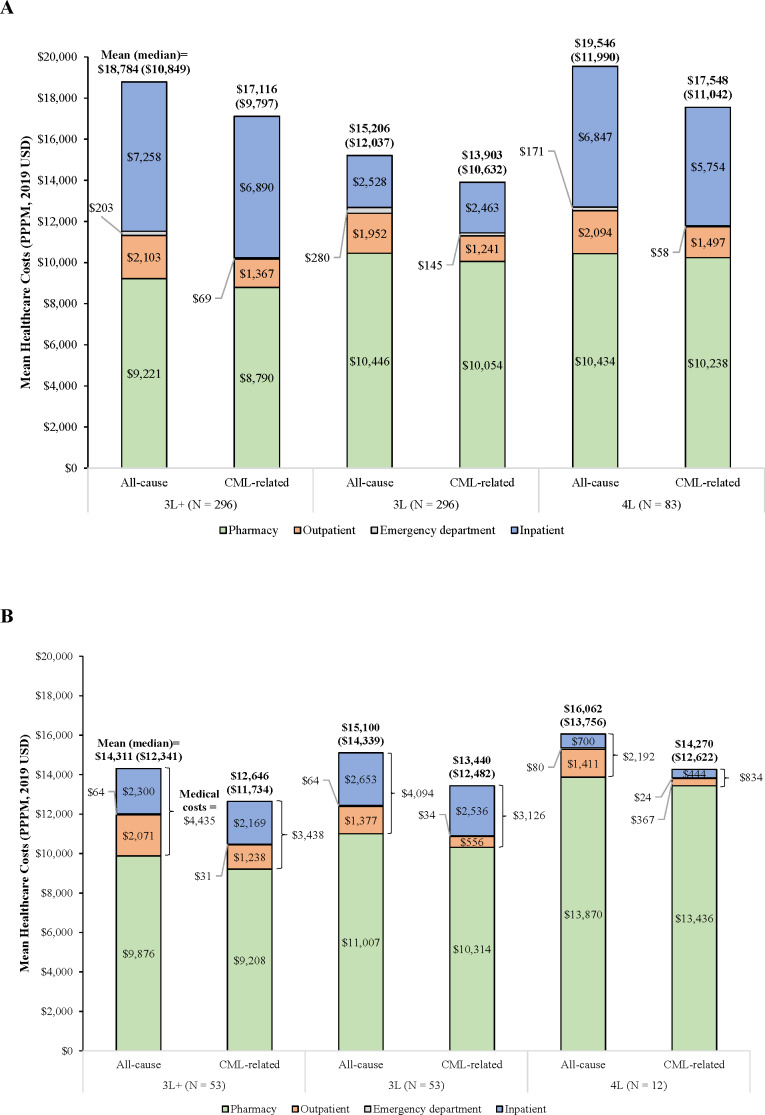
Healthcare Costs Stratified by Line of Therapy Among (**A**) Commercially Insured Patients and (**B**) Medicare Beneficiaries Abbreviations: CML, chronic myeloid leukemia; PPPM, per patient per month; USD, US dollars; 3L, third line; 3L+, third line or later; 4L, fourth line.

Mean (median) total all-cause healthcare costs were \$18 784 (\$10 849), \$15 206 (\$12 037), and \$19 546 (\$11 990) PPPM during 3L+, 3L, and 4L, respectively (Figure 4A), with pharmacy representing a substantial proportion of costs. The observed difference in total all-cause healthcare costs between the 3L and 4L was primarily driven by IP costs (mean [3L] = \$2528 PPPM, mean [4L] = \$6847 PPPM). Regardless of the line considered, at least 84% of these costs were CML-related. After excluding HSCT costs, mean (median) total all-cause costs were \$13 399 (\$10 708) PPPM in 3L+, \$14 670 (\$12 037) PPPM in 3L, and \$15 073 (\$11 517) PPPM in 4L (data not shown). Among patients who underwent HSCT after initiating 3L+ (6.8%), the mean (median) HSCT-related costs were \$222 498 (\$152 770; data not shown).

**Medicare-insured population**: Medicare-insured patients were observed over a median follow-up of 39 months during 3L+. Patients had 1.0 IP stays per year, of which 85.1% were CML-related (Figure 3B). During the same period, the annual IR for IP days, OP days, and ED visits were 10.3, 61.9, and 1.5, respectively. Largely similar annual IRs were observed during 3L and 4L.

Mean (median) total all-cause healthcare costs were \$14 311 (\$12 341) PPPM in 3L+, \$15 100 (\$14 339) PPPM in 3L, and \$16 062 (\$13 756) PPPM in 4L, with pharmacy driving the total costs. Across all lines, at least 88% of total all-cause healthcare costs were CML-related (Figure 4B). After excluding HSCT costs, the mean total all-cause healthcare costs were \$13 342 (\$12 341) PPPM in 3L+, \$15 013 (\$14 339) PPPM in 3L, and \$16 056 (\$13 747) PPPM in 4L (data not shown). The proportion of patients who underwent HSCT after initiating 3L therapy was low. Among 16 patients who underwent HSCT after initiating 1L therapy, the mean (median) HSCT-related costs were \$126 607 (\$104 528; data not shown).

## DISCUSSION

This study is the first to document the treatment patterns and economic outcomes of patients with CML who received 3L+. In this retrospective cohort study of patients with CML who received a 3L+, patients generally cycled between a 1G and 2G TKI through the first 3 lines of therapy and remained on each line for a short period of time (ie, median \~5-10 months). In addition, 3G TKI (ie, ponatinib) was infrequently used in later lines, as was HSCT. Average total all-cause healthcare costs were greater during 4L than 3L; this difference was mainly driven by IP costs, particularly among commercially insured patients. Given the rarity of HSCT among included patients, these costs remained largely similar in the sensitivity analysis that excluded HSCT costs.

The algorithm used to identify lines of therapy and treatment sequences leveraged several features of algorithms used in previous studies,^25,32–35^ although some were improved and others were added to better reflect real-world clinical practice. A new element is the fact that treatment gaps followed by the reinitiation of the same TKI were considered as treatment interruptions rather than a new line to better reflect the presumed clinical intention underlying these treatment patterns. Furthermore, the algorithm accounted for a comprehensive set of events that marked the end of a line of therapy, including HSCT and the initiation of a chemotherapy regimen not listed for CML-CP; most previous studies did not account for these events.^22,32,34–36^ These methodological improvements thus build on the existing literature.

Some elements from the resulting treatment patterns observed in this study highlight the unmet medical needs of patients who receive a 3L+. In the current study, the median duration of each line ranged between approximately 5 to 10 months, which is significantly shorter than most previous estimates for the 1L (range [median], 18.2-39.8 months)^22,37,38^ and 2L (11.0-22.4 months).^22,38^ Although reasons behind TKI switch are not available in claims, based on observations from the data for this study, the low use of ponatinib, the relative rarity of HSCT, and the relatively short duration of treatment may suggest that TKI resistance may not be a predominant driver of treatment switching in the current study. Additional factors such as the lack of a suitable donor or poor patient health (eg, two-thirds of the Medicare population was likely unfit for HSCT) may explain the infrequent use of HSCT, while pre-existing comorbidities like cardiovascular conditions could explain the infrequent use of ponatinib.^39^ Consistent with this hypothesis, prior studies have shown that intolerance is the most common reason for treatment switching.^22,36,40^

Although comparisons across studies must be carefully interpreted, the costs herein observed for patients who received a 3L+ were seemingly higher than those reported in previous studies that focused on 1L or 2L. These studies reported average total all-cause healthcare costs ranging between approximately \$10 000 to \$13 000 PPPM,^25,41^ whereas the current study reported costs ranging between \$15 206 PPPM (for the 3L) and \$19 546 PPPM (for the 4L). This apparent difference was particularly large for medical costs, especially in commercially insured patients. In this population, medical costs ranged from approximately \$1900 to \$2800 PPPM in studies that focused on the 1L and 2L compared with \$4760 (for the 3L) and \$9564 (for the 3L+) in the current study.^25,34,41^ Further, all-cause medical costs accounted for 31.3% (for 3L) to 50.9% (for 3L+) of total all-cause healthcare costs among commercially insured patients, whereas this proportion was only 18.8% to 29.3% in previous studies in 1L or 2L^25,34,41^ Given these substantial medical costs, new agents with improved tolerability and efficacy profiles may result in significant cost savings. Altogether, these results highlight the substantial economic burden of patients who receive a 3L+. It is worth noting that most patients included in the present study initiated a 3L+ prior to the introduction of generic imatinib in 2016, which translated into lower costs for patients initiated on this agent. However, no patients in the Medicare population and only 11.1% of those in the commercially insured population initiated their index TKI in 2016 or later. Further, as anticipated, imatinib was primarily used as 1L agent in the current study. As a result, the use of generic imatinib should have a minimal impact on the results of the current study for later lines for CML.

### Limitations

The results of the current study should be interpreted in light of certain limitations. First, the databases used for this study did not contain laboratory testing results. As a result, data on CML phase (eg, percentage of blast cells), disease severity (eg, Eastern Cooperative Oncology Group performance status, or tumor genotype), and molecular/cytogenetic response to treatment could not be assessed. However, the present study assumed that less than 5% of patients were in the accelerated phase or blast crisis. Second, reasons for initiating/terminating a line of therapy (eg, intolerance, resistance) were not available in the database. However, prior research has demonstrated that the most common reason for switching treatment is intolerance.^36^ Third, as the number of patients identified in the SEER-Medicare analysis was small, the study findings from the Medicare perspective may not be generalizable to the overall US Medicare population; further research with a larger sample size is warranted. Fourth, the study was also subject to common limitations of claims database analyses. Indeed, claims databases only record diagnostic and procedure codes that are recorded for reimbursement purposes. Furthermore, the presence of pharmacy claims for a filled prescription does not guarantee the actual consumption of the medication by the patient. Fifth, retrospective databases may contain coding errors or data omissions. Lastly, this study included patients with CML with commercial, Medicare Supplemental, and Medicare insurance coverage and thus may not be representative of patients with Medicaid or no insurance reaching 3L+.

## CONCLUSIONS

This study characterized patients with CML who received at least 3 lines of therapy, an understudied population withsignificant unmet medical needs. Regardless of the study population (ie, commercially or Medicare-insured patients), few patients included in this study underwent HSCT. In addition, patients rapidly cycled through multiple lines, highlighting the clinical challenges faced by clinicians to treat these patients. Total all-cause healthcare costs increased from 3L to 4L; in the commercially insured population, this increase was predominantly driven by higher IP costs, suggesting potential cost savings with safer and more efficacious drugs. Altogether, these findings support the need for continued drug research and new and more efficacious treatment options to improve patients’ outlook and reduce costs in this difficult-to-treat population.

### Disclosures

E.L.A. has provided paid consulting services to Bristol Myers Squibb, Pfizer, Novartis Pharmaceuticals Corporation, Jazz Pharmaceuticals, and Abbvie and has been paid to participate in a speakers’ bureau for Novartis Pharmaceuticals Corporation, Jazz Pharmaceuticals, and AbbVie. R.M. is an employee of Novartis Pharmaceuticals Corporation. D.L.V., C.R., A.G., and E.Q.W. are employees of Analysis Group, Inc, a consulting company that has provided paid consulting services to Novartis Pharmaceuticals Corporation, which funded the development and conduct of this study. P.P. was an employee of Novartis Pharmaceuticals Corporation at the time of the conduct of the study.

### Author Contributions

All authors (E.L.A., R.M., P.P., D.L.V., C.R., A.G., and E.Q.W.) contributed to the design of the study and interpretation of the data. D.L.V., C.R., and E.Q.W. contributed to the data acquisition. D.L.V., C.R., and A.G. contributed to the data analysis. All authors (E.L.A., R.M., P.P., D.L.V., C.R., A.G., and E.Q.W.) critically revised the draft manuscript and approved the final content.

### Data Availability

The IBM® MarketScan® Research Databases and SEER-Medicare databases are not publicly available.

### Ethical Compliance

For the use of SEER-Medicare data, the study received an IRB exemption from the Western Copernicus Group Institutional Review Board (WCGIRB). Per data user agreement, due to the cell suppression policy of the Centers for Medicare & Medicaid Services, cell values of less than 11 cannot be displayed for results from SEER-Medicare data.

## Supplementary Material

Online Supplementary Material
