# Supplementary material for: Chronic Myeloid Leukemia: Part I—Real-World Treatment Patterns, Healthcare Resource Utilization, and Associated Costs in Later Lines of Therapy in the United States
Source: J Health Econ Outcomes Res. 2022 Aug 4;9(2):19–29. doi: 10.36469/001c.36975 (PMC9352872; doi:10.36469/001c.36975)
Supplement: Online Supplementary Material [file jheor_2022_9_2_36975_95909.pdf]

Journal of Health Economics and Outcomes Research

Online Supplementary Material

Chronic Myeloid Leukemia: Part I—Real-World Treatment Patterns, Healthcare Resource Utilization, and Associated Costs in Later Lines of Therapy in the United States. *JHEOR*. 2022;9(2):19-29. doi:10.36469/jheor.2022.36975

Figure S1: SEER-Medicare Linkage

Figure S2: Study Design Scheme

This supplementary material has been provided by the authors to give readers additional information about their work.

This is an open-access article distributed under the terms of the Creative Commons Attribution 4.0 International License (CCBY-4.0). View this license's legal deed at http://creativecommons.org/licenses/by/4.0 and legal code at http://creativecommons.org/licenses/by/4.0/legalcode for more information.

Figure S1. SEER-Medicare Linkage

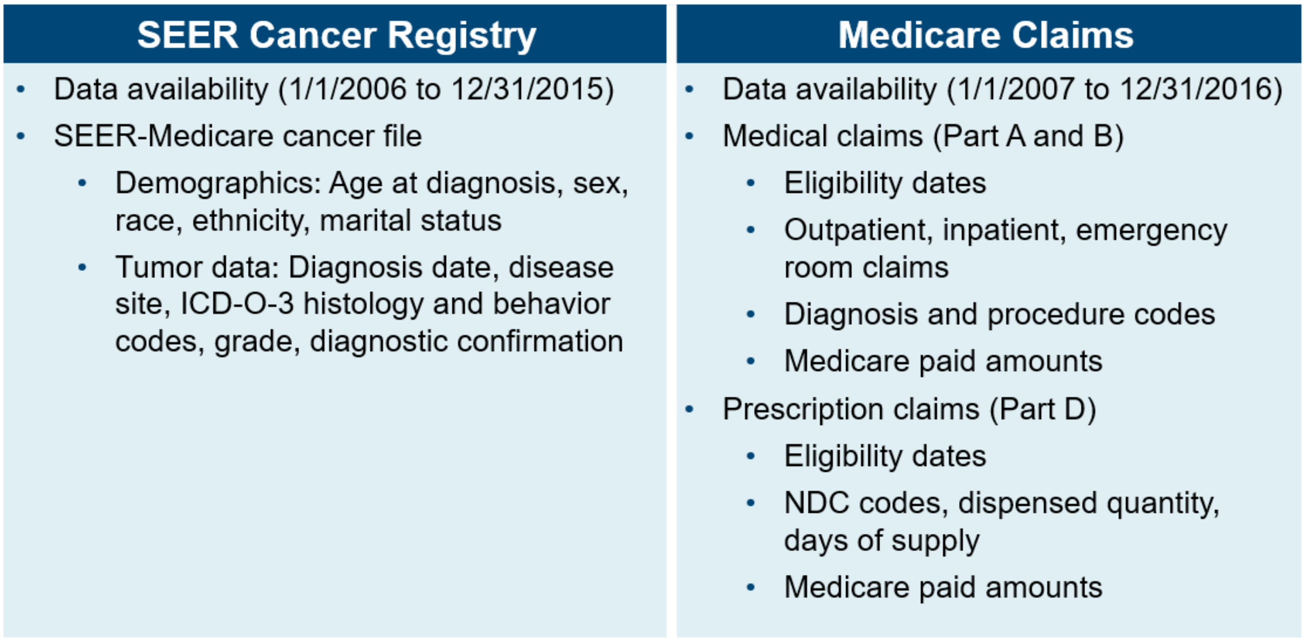

Abbreviations: ICD-O-3; *International Classification of Diseases for Oncology, Third Edition;* NDC, National Drug Code; SEER, Surveillance, Epidemiology, and End Results Program.

Figure S2. Study Design Scheme

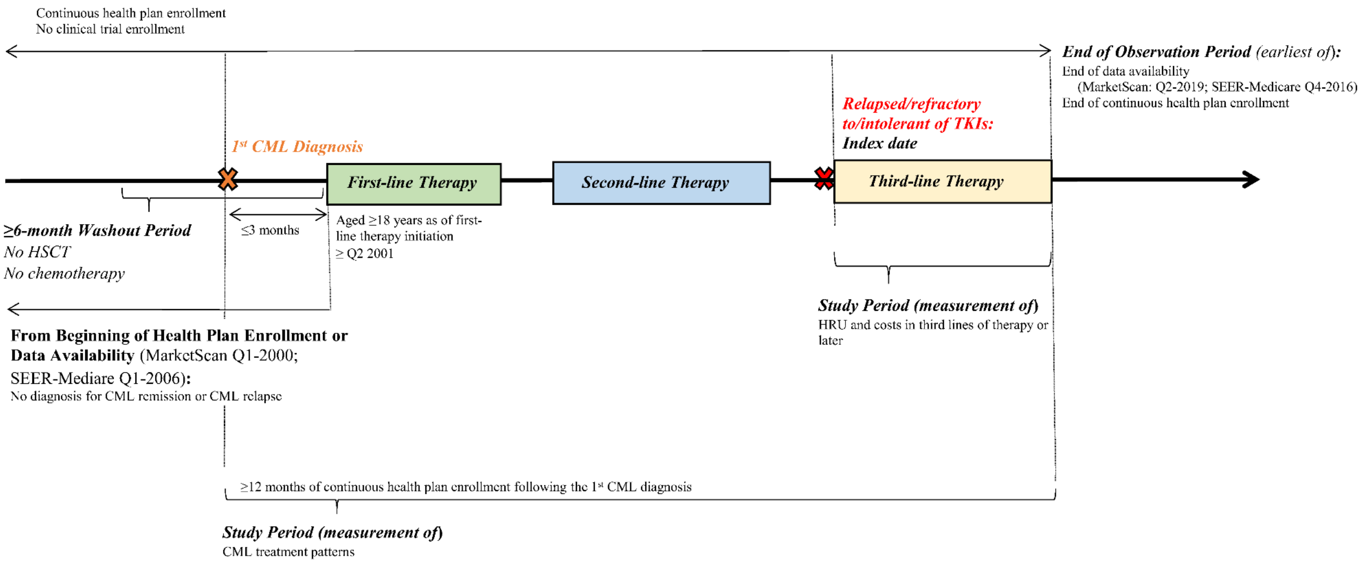

Abbreviations: CML, chronic myeloid leukemia; HRU, healthcare resource utilization; HSCT, hematopoietic stem cell transplantation; TKI, tyrosine kinase inhibitor.
